# The Impact of eSports Industry Knowledge Alliances on Innovation Performance: A Mediation Model Based on Knowledge Sharing

**DOI:** 10.3389/fpsyg.2022.902473

**Published:** 2022-05-02

**Authors:** Longfei Yue, Yiwen Zheng, Meng Ye

**Affiliations:** ^1^School of Physical Education, Sichuan University, Chengdu, China; ^2^Faculty of Education, University of Macau, Macau, Macao SAR, China; ^3^Business School, Sichuan University, Chengdu, China

**Keywords:** knowledge sharing, innovation performance, member ability, member relationships, eSports industry, knowledge alliances

## Abstract

This study investigates the associations among member ability, member relationships, knowledge sharing, and innovation performance in eSports industry knowledge alliance. A survey strategy and purposive sampling were applied, and the analysis was conducted on a sample of 311 senior managers from the China eSports Association. The hypotheses were tested using SPSS 24.0 software and AMOS 24.0 software. This study shows that member ability and member relationships have both a direct and indirect effect on innovation performance. Firstly, member ability, member relationships, and member knowledge sharing significantly impact the innovation performance of eSports industry knowledge alliances. Secondly, member knowledge sharing plays a mediating role in the effect of member ability and membership relationship on innovation performance. This pioneering article explores the interaction mechanisms between member ability, member relationships, and innovation performance in eSports industry knowledge alliance. The research results are conducive to the development of the eSports industry toward deep integration and sustainable development and provide a reference for similar knowledge-intensive enterprise alliance behaviors.

## Introduction

eSports first originated in the 1990s. As an emerging competitive sport, eSports have developed into electronic game competitive sports with the same spirit as that of modern competitive sports (Heere, 2018). It is generally believed that the eSports industry plays a significant role in stimulating economic development and solving the issue of unemployment (Kim et al., 2020). The eSports industry chain attracts multiple parties to participate, and the establishment of strategic alliances is a relatively common form of organization in this industry. With the advent of the knowledge economy, knowledge has replaced traditional resources as a critical hidden asset and is an essential source of sustainable competitive advantage for enterprises (Drucker, 1999; Popkova, 2019). In the context of the rapid development of knowledge management theory, the research on strategic alliance theory has gradually evolved into a new concept–the knowledge alliance (Morrison and Mezentseff, 1997; Schoenmakers and Duysters, 2006). A knowledge alliance is a partnership in which enterprises or other institutions cooperate closely. Its essence is to create new knowledge and carry out knowledge transfer jointly. The knowledge alliance is not a collaborative relationship formed to expand production and sales but rather one to pay more attention to low-cost knowledge exchange, sharing, and innovation among different organizations within the alliance. Through knowledge alliances, companies can gain knowledge innovation capabilities (Zhang et al., 2019; Zhao et al., 2021). Innovation is an inexhaustible driving force for developing knowledge alliances (Udriyah et al., 2019; Distanont and Khongmalai, 2020).

Choosing the right knowledge partner for an eSports industry knowledge alliance becomes the key to its success. The selection of members, especially core members, is related to realizing all strategic goals and benefits of the union. Member capabilities and relationships have an enormous impact on industry alliances (Todeva and Knoke, 2005; Rezazadeh and Nobari, 2018). Following the characteristics of eSports knowledge innovation, the present research evaluates members’ capabilities from two aspects: knowledge specificity and knowledge innovation ability. The closeness of the relationship between alliance members is assessed from two factors: cooperation spirit and trust. Knowledge sharing among members is the basis for knowledge innovation in eSports industry knowledge alliances (Li et al., 2022). Teece (1986) mentioned that it is difficult for any organization to obtain all knowledge resources and that all knowledge resources must be scattered among various organizations. Therefore, acquiring knowledge from other organizations through limited knowledge sharing has become the best choice for enterprises. Hamel (2005) found that the primary purpose and motivation for establishing alliances between enterprises is to share knowledge resources through mutual learning. Knowledge sharing can be understood as the flow of knowledge from one organization to another. However, alliance members need to use their absorptive capacity to digest and absorb the newly acquired knowledge and further integrate it into their knowledge system, transforming it into new knowledge (Arora et al., 2021).

With the construction of knowledge alliances, enterprises can solve the problem of insufficient internal knowledge capital resources, enabling them to form cooperation, mutual assistance, and mutual supplementation to achieve the joint acquisition and effective use of knowledge capital. However, whether the emergence of knowledge alliances among eSports companies can play the role of knowledge innovation and promote the development of new technologies remains to be further studied. Based on this, this article takes knowledge governance as the theoretical basis and examines mainly whether the relationship between members and the ability of members in eSports industry knowledge alliances affects the innovation performance of alliances through knowledge sharing.

## Theoretical Model and Hypotheses

### Knowledge Governance Theory

The idea of knowledge governance starts from the vast divergence and continuous debate between transaction cost theory and enterprise knowledge theory. In the past 30 years, the vigorous rise of knowledge movement and knowledge management indicates that human society is entering the development stage of knowledge society from industrial society. The concept of knowledge governance proposed by Grandori (2001) replaces the original enterprise theory. He believes that its definition should govern knowledge exchange, transfer, and sharing within and outside the organization and the coordination mechanism of knowledge nodes. Foss et al. (2003) propose another definition: knowledge governance is the optimization of knowledge acquisition, construction, sharing, and distribution by selecting or influencing formal organizational mechanisms and structures. Antonelli (2005) believes that knowledge governance is an administrative form of knowledge production and use through institutions, policies, corporate strategies, transaction types, and interactions. From the definitions given by the above scholars, it can be seen that knowledge governance is an activity that optimizes the acquisition, exchange, transfer, sharing, distribution, flow, innovation, and other forms of knowledge in enterprises to achieve the purpose of knowledge utilization and development of new knowledge. The research object of knowledge governance is how different organizational structures, incentive methods, contract methods, other rigid factors, psychological contracts, corporate culture, etc., have different effects on organizational knowledge management activities(Foss, 2007).

The eSports industry is a brand-new industrial form, and its development requires a corresponding management form. The e-sports industry alliance is a common intermediate organization form for e-sports enterprises. The development of eSports enterprises characterized by knowledge production, exchange, and utilization has different requirements from the traditional economy. The cross-border integration between industries is to achieve the extension of the industrial value chain through the mutual integration of existing industrial elements and resources. In this process, knowledge sharing among members is of great significance to the development of the Esports knowledge alliance.

### Member Ability and Alliance Innovation Performance

The choice of alliance partners has always been one of the hotspots of industry alliance research. The choice of which members to cooperate with has an enormous impact on industry alliances. In alliance formation, the primary consideration for members is the complementarity of resources between associations (Furlotti and Soda, 2018; O’Dwyer and Gilmore, 2018). Companies that have vital resources that other members do not have are more attractive to those other members. This essential resource can be a specific technology or successful management experience. Harrison et al. (2001) pointed out that if the resources of alliance members are too similar, then the performance of that alliance is far inferior to that of those alliances with different resources that can complement one another. At the same time, when a company is in a highly uncertain environment, identifying companies with complementary resources with which to establish partnerships to reduce the impact of environmental changes on the company becomes increasingly necessary. The importance of membership capabilities to the success or failure of industry alliances has been confirmed by the extant research. Drawing on the absorptive capacity perspective, Lin et al. (2012) argued that firms with a high level of such capacity seem to benefit more from their alliances than those with a low level of such ability. Argote and Ingram (2000) suggested that corporate learning capabilities affect alliance innovation, while Cummings and Teng (2003) said that companies’ alliance experience and qualifications affect alliance innovation. Moreover, Makri and Lane (2010) suggested that corporate organizational structure, partner selection, and similarity between companies affect alliance innovation. Since the innovation activities of the eSports industry require many resources and a great deal of time, once an alliance’s knowledge innovation fails, its members may lose valuable development opportunities. Therefore, eSports knowledge alliances must carefully select members to reduce risks as much as possible. Members who are too weak are not conducive to the development of eSports knowledge innovation. In this study, based on previous studies, members’ ability is evaluated in terms of knowledge exclusivity and knowledge innovation ability.

The knowledge exclusivity of members refers to the number of standards and patents owned by an organization. The adequate management of the intellectual property is critical to sustaining competitive advantage and managing outbound open innovation, which describes the inside-out flows of knowledge and technology (Grimaldi et al., 2021). Intellectual property and knowledge management practices positively correlate with innovation activities (Law et al., 2021). The results of Roh et al. (2021) revealed that a firm’s intellectual property rights and government support significantly affect open, green process, and green product innovation. The innovation of eSports knowledge covers many aspects, the most valuable of which is eSports content and technology, and the most urgently needed is the innovation of eSports industry standards (Kim et al., 2020). In eSports industry knowledge alliances, related intellectual property rights can be divided into core and marginal categories. A core intellectual property right is highly related to the alliance’s knowledge innovation direction. Core intellectual property plays a significant role in the knowledge innovation process of partnerships. If the core intellectual property is lacking, then the knowledge innovation of the alliance ends. Members with core intellectual property rights can occupy a dominant position in the alliance and have a vital influence on its development (Wang et al., 2021). The success of an alliance’s knowledge innovation further enhances the competitiveness of core members.

The knowledge innovation ability of members examines mainly the organization’s knowledge stock, whether the enterprise has carried out knowledge innovation, and the frequency, quantity, and level of such innovation. In the innovation process of eSports industry knowledge alliances, the learning ability of members is crucial. In the highly technical and fast-developing eSports industry, any enterprise can quickly adapt to the development of the situation and absorb and learn knowledge quickly. The research of Gianmario and Davide (2003) confirmed that the innovation ability of members promotes the overall innovation ability of the alliance. Jin and Yun (2013) studied the mechanism of technological innovation in enterprise management innovation and suggested that management innovation is the guarantee and prerequisite for technological innovation. Suppose a particular enterprise in an alliance has a high level of innovation. It affects its innovation and positively impacts other members, thereby improving overall innovation performance. The research results of Bai et al. (2021) demonstrated that organizational learning capacity positively affects the innovation performance of freight logistics services. From the above analysis, it can be seen that the partner selection of an industry alliance is one of the essential dimensions that affect the performance of eSports industry alliances. Based on this result, combined with the knowledge innovation characteristics of the eSports industry, the paper puts forward the following hypotheses:

***H1:** Knowledge exclusivity has a significantly positive impact on alliance innovation performance.*

***H2:** Knowledge innovation ability has a significantly positive impact on alliance innovation performance.*

### Member Relationship and Alliance Innovation Performance

How the relationship between members affects the performance of industry alliances is also one of the critical areas to which researchers have paid attention. The relationship between knowledge alliance members is divided into many levels, with different researchers conducting research from different perspectives. Robert et al. (1998) proposed that the mutual relationship of alliance members can be described in terms of investment level, return, trust, uncertainty, etc. According to the characteristics of eSports knowledge innovation, from the perspective of the closeness of alliance members, the level of the relationship between eSports industry knowledge alliance members is mainly reflected in two aspects: cooperative spirit and trust level.

Boh et al. (2020) found that corporate investors with broad investment experience strengthen a firm’s environmental scanning abilities, enhancing innovation performance by increasing the number of external cooperation activities in which the firm engages. Choi and Choi (2021) found that vertical R&D cooperation positively affected overall industry performance, especially on service and marketing performance.

eSports knowledge comes from different fields, and the eSports industry is constantly absorbing innovations from other areas and applying them to eSports. Suppose a good member cooperative relationship can be formed in an eSports industry knowledge alliance. In that case, the knowledge achievements of different subjects can be used to promote the effective development of eSports knowledge innovation activities. Suppose a good member cooperative relationship can be formed in an eSports industry knowledge alliance. In that case, the knowledge achievements of different subjects can be used to promote the effective development of eSports knowledge innovation activities. The effect of member partnership on the performance of eSports industry knowledge alliances is more manifested as a process effect. Suppose the spirit of cooperation among members is poor. In that case, the knowledge innovation activities of the eSports industry knowledge alliance are unable to operate, resulting in the alliance’s failure. In contrast, if there is a spirit of cooperation among alliance members, the knowledge innovation process between associations is smoother.

The coexistence of competition and cooperation is the primary feature of the membership of an industrial alliance. Although establishing an industrial partnership promotes knowledge sharing, the relationships between industrial alliance members cannot be regarded as only cooperative. Harrigan (1986) suggested that industry alliances need to formulate rules or sign contracts to regulate and constrain members’ behavior. When dealing with the relationship between members of a knowledge alliance, trust among members is extremely important. At the same time, Ring and Van de Ven (1994) suggested that establishing a trusting relationship between individuals is the key to alliance success. Later, Birnberg (1998) came to the same conclusion, meaning that trust can have the same effect as a control. In his analysis of alliance trust, he proposed that the analysis results come from two assumptions: first, alliance members believe that default makes them lose more, and second, alliance members hope to form a set of reciprocity standards. Adobor (2005) suggested that the establishment of sufficient trust among alliance partners is the key to alliance success and analyzed the generation mechanism of trust in the context of cooperation. Trust is the basis and premise of collaboration and can also improve the efficiency of communication and collaboration among members and reduce the dependence on formal rules and regulations (Poppo and Zenger, 2002). Le and Lei (2018) found a mediating role of trust in stimulating the relationship between transformational leadership and knowledge sharing processes. The results of Arroyave et al. (2020) showed that firms that value cooperation with universities develop a more comprehensive range of environmental innovations and increase their sales and benefits.

As an informal organization, an eSports industry knowledge alliance cannot establish a power relationship between subordinates as can a single organization. Therefore, effective collaboration is more critical to the coalition. The establishment of mutual trust and cooperation between different subjects can better stimulate members’ creativity through information exchange to discover opportunities for innovation and obtain innovative results. Accordingly, the paper proposes the following hypotheses:

***H3:** Cooperative spirit has a significant positive impact on alliance innovation performance.*

***H4:** Trust level has a significant positive impact on alliance innovation performance.*

### Knowledge Sharing and Alliance Innovation Performance

The theory of knowledge holds that knowledge is an essential resource for maintaining the competitive advantage of market players (Grant and Baden-Fuller, 1995). Enterprises must effectively acquire and develop knowledge. In addition to integrating one’s internal knowledge, acquiring external knowledge is also essential for an enterprise. Organizational learning theory suggests that learning from other organizations is crucial for enterprises acquiring knowledge. Through practical learning, enterprises can develop new knowledge and expand the depth and breadth of such knowledge (Lane et al., 2001). An important consideration for members in joining a knowledge alliance is to share and exchange knowledge with partners and apply it to their development process to make up for their knowledge deficiencies at a relatively small cost. Obtaining knowledge from other alliance members can enrich the knowledge reserve of members and help them innovate knowledge and seize new market opportunities (Kim et al., 2011). Most scholars have suggested that knowledge sharing positively impacts innovation performance. Using hierarchical multiple regression and moderated multiple regression methods, the results from a survey of 236 firms in China indicated significant positive relationships among collaborative innovation activities, knowledge sharing, collaborative innovation capability, and firm innovation performance (Wang and Hu, 2020). Mardani et al. (2018) found that knowledge creation, integration, and application facilitate innovation and performance. Muhammed and Zaim (2020) found that the extent of employees’ engagement in knowledge sharing behavior has a positive impact on organizations’ knowledge management success, which, in turn, can positively affect organizations’ innovation performance. Tassabehji et al. (2019) investigate knowledge sharing and its contribution to firm innovation performance improvements. Results from a survey of 236 firms in China indicated significant positive relationships between collaborative innovation activities, knowledge sharing, collaborative innovation capability, and a firm’s innovation performance. Moreover, knowledge sharing is expected to play a partial mediating role in the relationships between collaborative innovation activities and the firm’s innovation performance (Wang and Hu, 2020). Knowledge sharing and innovation strategy fully mediate the relationship between outside-in OI and innovation performance (Bagherzadeh et al., 2019). The result of Hanifah et al. (2021) shows that knowledge sharing significantly impacts firm innovation performance. Based on the above analysis, this paper puts forward the following hypothesis:

***H5:** Member knowledge sharing has a significant positive impact on alliance innovation performance.*

### Mediating Effect of Knowledge Sharing

In the era of the knowledge economy, knowledge, especially tacit knowledge, is the key to the core competitiveness of enterprises. Knowledge sharing can effectively integrate mutually complementary resources to rapidly respond to the market and improve competitive advantages (Wang and Noe, 2010; Jiang and Chen, 2021).

Knowledge sharing can be understood as the process of knowledge dissemination among individuals or organizations to further absorb and internalize it into their knowledge and, on this basis, carry out further innovation to realize value creation. The knowledge management theory shows that only through a wider range of mutual communication, learning and sharing can the utilization and value-added effect of knowledge be better. The research of Singh et al. (2021) suggested that top management knowledge value and knowledge sharing practices influence open innovation, which, in turn, affects organizational performance.

Knowledge-sharing behavior has long been regarded as the most crucial link between knowledge management factors. Knowledge sharing is an indicator of knowledge management and organizational learning effectiveness (Yang and Xu, 2021). Lee (2001) suggested that knowledge sharing at different levels between firms and within firms has different effects on innovation performance. Song et al. (2015) examined servant leadership as a precursor to a knowledge-sharing climate. They demonstrated the mediating role of such a knowledge-sharing climate in the relationship between servant leadership and team performance. Al-Husseini et al. (2021) examined the linkages among transformational leadership, knowledge sharing, and innovation in higher education. A positive direct impact was found in transformational leadership, knowledge sharing, and innovation. Moreover, knowledge sharing was identified as a mediator between transformational leadership and innovation.

Accordingly, the paper proposes the following hypotheses:

***H6:** Knowledge sharing mediates the effect of knowledge exclusivity on innovation performance.*

***H7:** Knowledge sharing mediates the effect of learning innovation ability on innovation performance.*

A good partnership can promote knowledge sharing among enterprises, and there is also a positive relationship between knowledge sharing and innovation performance. Knowledge collaboration can improve the efficiency of knowledge flow and sharing and is also a meaningful way to generate value-added knowledge (Cheng and Chang, 2020). Knowledge sharing is a mediator between collaborative culture and innovation capability (Yang et al., 2018).

Inkpen (1998) pointed out that members take a cautious attitude toward knowledge sharing, but to achieve the alliance’s goals, alliance members can only share knowledge. In addition, member trust helps promote the knowledge transfer of the alliance. Moreover, the above author pointed out that the accessibility of alliance knowledge, including the trust among alliance members, the degree of knowledge protection, the degree of tacit knowledge, and the past development history of alliance members, are the main factors that affect alliance knowledge sharing. Delbufalo (2012) suggested that this kind of trust is extremely important to the willingness to share knowledge among alliance members. The results of the (Ogunmokun et al., 2020) revealed that both knowledge sharing behaviour mediates the positive effect of propensity to trust on service innovation. Accordingly, this paper proposes the following hypotheses:

***H8:** Knowledge sharing mediates the effect of cooperative spirit on innovation performance.*

***H9:** Knowledge sharing mediates the effect of trust level on innovation performance.*

**FIGURE 1:**
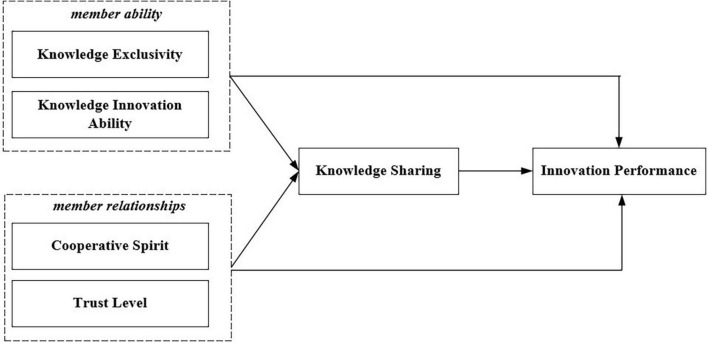
Research model of the study.

In the process of knowledge alliance innovation, alliance members need to cooperate with different types of subjects to combine different kinds of innovation resources (Steensma and Corley, 2000). The members of eSports industry knowledge alliances must collaborate with other members, gain new knowledge from other members, and jointly invest in eSports knowledge innovation through knowledge sharing. This paper constructs the innovation performance relationship model of eSports industry knowledge alliances from the perspective of the factors of eSports industry member ability, member relationships, and member knowledge sharing, as shown in Figure 1.

## Materials and Methods

This study is cross-sectional, and a structured questionnaire was used to evaluate quantitative data. A total of 23 items were employed to develop a questionnaire for variable analysis. The questionnaire takes the form of a commissioned survey. Participants are senior managers of eSports competition organizers, eSports content producers, eSports operators, eSports media, eSports equipment developers and manufacturers, eSports participants, and eSports management departments. Through the recommendation of the Sichuan eSports Association, an electronic questionnaire is distributed among alliance members in 22 provinces, autonomous regions, and municipalities affiliated with the China eSports Association. A total of 400 electronic questionnaires were distributed, and 346 questionnaires were recovered, for a recovery rate of 86.5%. 35 invalid questionnaires are eliminated, and 311 valid questionnaires are finally obtained for an effective rate of 90%. The reliability and validity tests of the measurement scale and the whole model were tested using SPSS 24.0 software and AMOS 24.0 software. As a result, previous research measurements were used in this study to assess all the constructs of the current model.

### Variable Measurement

Researchers can develop measurement scales using deductive methods based on theoretical support and a clear understanding of the connotation and structure of constructs (Hinkin, 1995). This paper refers to the existing literature to design measurement items for each variable. The scale items are revised according to the interviews conducted with managers of eSports enterprises and industry associations in eSports industry knowledge alliances, as well as experts and scholars in related research fields. All variables in the questionnaire are scored using a 5-point Likert scale, with “completely disagree,” “partially disagree,” “uncertain,” “partially agree,” and “completely agree” corresponding to scores of 1–5, respectively. The questionnaire is divided into two parts. The first part is the basic information of respondents, including gender, age, working time in the eSports industry, and the type of eSports organization for which they work. The second part is the measurement items of the main variables involved in this research, namely, alliance member ability (knowledge specificity and knowledge innovation ability), alliance member relationship (cooperative spirit and trust level), alliance member knowledge sharing, and alliance innovation performance, for a total of 6 variables and 23 items.

In this study, the ability of alliance members involves two dimensions: knowledge specificity and learning and innovation ability. According to Hitt et al. (2012), this study sets 4 items for the measurement of knowledge specificity and learning innovation ability. Alliance membership in this study involves two dimensions: cooperative spirit and trust level. According to the research of Chen and Yang (2014), four items are set to measure cooperation spirit and trust level. This paper refers to the study of Ipe (2003) and selects three items to measure the knowledge-sharing behavior of alliance members. Drawing on the views of scholars such as Brouwer and Kleinknecht (1999), combined with in-depth interviews, this paper sets up four items for the measurement of alliance innovation performance and verifies the independence of the above indicators.

### Descriptive Analysis

The study sample is 66% male and 34% female. In terms of age, the 20-to-30-year-old age group is the majority, accounting for 45.02% of the sample, followed by the 31-to-40-year-old age group, accounting for 25.72%, the under 20-year-old age group, accounting for 12.54%, the 41-to-50-year-old age group, accounting for 10.93%, and the over-50-year-old age group, accounting for 5.79%. In terms of working time in the eSports industry, the proportion of those working less than 1 year is the largest, accounting for 39.55%; the second is those working 1–3 years, accounting for 38.91%; and the third is those working more than 6 years, accounting for 12.54%. The lowest is those working 4–6 years, accounting for 9.00%. In terms of the types of eSports organizations, eSports participants and eSports management departments account for the largest proportions, at 18.33% and 15.43%, respectively. The remaining eSports organizations are eSports competition organizers, eSports operators, educational institutions, eSports media, scientific research institutions, eSports content producers, and eSports equipment developers and manufacturers, the proportions of which are 12.86%, 11.58%, 11.58%, 10.61%, 8.36%, 5.79%, and 5.47%, respectively.

### Reliability Test

In this study, the average score of the six factors is used as the variable score. The mean, standard deviation, and pairwise Pearson correlation coefficient of the variables are calculated. The results are shown in Table 1, in which the Cronbach’s α reliability coefficient of the scale is in parentheses on the diagonal.

**TABLE 1:** Correlation analysis and reliability test results of each variable.

		Mean	SD	1	2	3	4	5	6	7	8	9	10
1	Gender	1.39	0.49										
2	Age	2.52	1.03	–0.035									
3	Working time	1.95	0.99	–0.069	0.157**								
4	Organization type	5.15	2.52	0.019	–0.057	–0.158**							
5	Knowledge exclusivity	4.11	0.73	0.009	–0.044	0.026	0.018	(0.614)					
6	Learning innovation ability	3.84	0.79	–0.083	–0.117*	–0.021	0.026	0.312**	(0.607)				
7	Cooperative spirit	4.00	0.79	–0.029	–0.229**	–0.033	0.013	0.275**	0.458**	(0.630)			
8	Trust level	3.59	0.82	0.008	–0.196**	–0.122*	0.049	0.223**	0.291**	0.334**	(0.709)		
9	Knowledge sharing	3.83	0.95	–0.029	–0.182**	0.02	0.031	0.255**	0.304**	0.336**	0.270**	(0.705)	
10	Innovation performance	3.65	0.88	–0.064	–0.275**	–0.145*	0.014	0.148**	0.401**	0.386**	0.357**	0.402**	(0.714)

**p < 0.01, *p < 0.05.

From Table 1, it can be seen that the correlation between the main variables in this study reaches a significant level, which lays the foundation for further hypothesis testing. At the same time, Cronbach’s α reliability coefficient values of all scales are more significant than 0.6, meeting the statistical requirements. It should be noted that from the test results, the Cronbach’s α value of each scale is shown not to be high, possibly because there are fewer measurement items (3 or 4 items) for each variable in this study. Few items are used for measurement because it is difficult to collect data on the senior managers of enterprises or organizations related to eSports industry knowledge alliances. Using fewer items to measure is convenient to meet the statistical requirements of 5–10 times between the sample size and item size.

## Results

### Empirical Analysis of the Main Effects

#### Influence of Member Ability on Alliance Innovation Performance

The linear regression method tests the influence of the two dimensions of alliance member ability on innovation performance. Table 2 presents the test results of linear regression.

**TABLE 2:** Regression analysis of alliance member ability on innovation performance.

Independent variable	Dependent variable: innovation performance			
	M1	M2	M3	M4
Gender	–0.081	–0.082	–0.048	–0.050
Age	−0.261**	−0.254**	−0.217**	−0.217**
Working time	−0.109*	−0.114*	−0.107*	−0.108*
knowledge exclusivity		0.141*		0.029
Learning innovation ability			0.370**	0.361**
R^2^	0.093	0.113	0.227	0.477
ΔR^2^	0.093	0.020	0.134	0.135
ΔF	10.49**	6.79*	52.95**	26.56**

N = 311; **p < 0.01, *p < 0.05; two-tailed test.

From Table 2, it can be seen that knowledge specificity and learning innovation ability in M2 and M3 have a significant positive impact on innovation performance (β = 0.141, *p* < 0.05; β = 0.370, *p* < 0.01), so research Hypotheses H1 and H2 are verified. When knowledge specificity and learning innovation ability are entered into the regression model together, only learning and innovation ability are positively significant for the impact of knowledge technology innovation (β = 0.361, *p* < 0.01), but knowledge specificity is not (β = 0.029, n.s.). This finding shows that alliance members’ learning and innovation ability has a greater impact on the performance of alliance knowledge innovation.

#### Influence of Member Relationships on Alliance Innovation Performance

A linear regression method is used to test the influence of the two dimensions of alliance membership on innovation performance. Table 3 presents the regression test results.

**TABLE 3:** Regression analysis of Member relationship on innovation performance.

Independent variable	Dependent variable: innovation performance			
	M1	M2	M3	M4
Gender	–0.081	–0.068	–0.079	–0.070
Age	−0.261**	−0.183**	−0.205**	−0.158**
Working time	−0.109*	−0.110*	–0.081	–0.088
Cooperative spirit		0.338**		0.269**
Trust level			0.308**	0.226**
R^2^	0.093	0.201	0.183	0.246
ΔR^2^	0.093	0.108	0.090	0.153
ΔF	10.49**	41.46**	33.89**	30.84**

N = 311; **p < 0.01, *p < 0.05; two-tailed test.

Table 3 shows that both the spirit of cooperation and trust in M2 and M3 have a significant positive impact on innovation performance (β = 0.338, *p* < 0.01; β = 0.308, *p* < 0.01). Therefore, research Hypotheses H3 and H4 are verified. When the spirit of cooperation and the trust level is entered into the regression model together, the two effects are still significant (M4, β = 0.269, *p* < 0.01; β = 0.226, *p* < 0.01).

#### Impact of Member Knowledge Sharing on Alliance Innovation Performance

The linear regression method is used to test the effect of knowledge sharing among alliance members on alliance innovation performance. Table 4 presents the test results.

**TABLE 4:** Regression analysis of member knowledge sharing on innovation performance.

Independent variable	Dependent variable: innovation performance	
	M1	M2
Gender	–0.081	–0.069
Age	−0.261**	−0.191**
Working time	−0.109*	−0.127*
Knowledge sharing		0.367**
R^2^	0.093	0.223
ΔR^2^	0.093	0.130
ΔF	10.489**	51.198**

N = 311; **p < 0.01, *p < 0.05; two-tailed test.

Table 4 shows that knowledge sharing among alliance members in M2 significantly affects innovation performance (β = 0.367, *p* < 0.01). Therefore, research Hypothesis H5 is validated.

### Empirical Analysis of the Mediation Effect

#### Mediating Role Played by Member Knowledge Sharing in the Effect of Member Ability on Alliance Innovation Performance

Under the control of gender, age, and working time, the macro plug-in PROCESS3.3 in SPSS24.0 was used to test the research hypothesis. A linear regression method is used to test the mediating role of alliance member knowledge sharing in the influence of the two dimensions of alliance member ability on innovation performance. Table 5 presents the test results of linear regression.

**TABLE 5:** The mediating effect of member knowledge sharing on the impact of member ability on innovation performance.

Independent variable	Dependent variable: knowledge sharing		Dependent variable: innovation performance			
	M1	M2	M3	M4	M5	M6
Gender	–0.033	–0.014	–0.082	–0.070	–0.048	–0.046
Age	–0.190**	–0.154**	–0.254**	–0.191**	–0.217**	–0.173**
Working time	0.047	0.043	–0.114*	–0.128*	–0.107*	–0.121*
Knowledge exclusivity		0.175**	0.141*	0.053		
Learning innovation ability		0.231**			0.370**	0.289**
Knowledge sharing				0.354**		0.283**
R^2^	0.036	0.144	0.113	0.226	0.227	0.298
ΔR^2^	0.036	0.108	0.020	0.133	0.134	0.205
ΔF	3.87*	19.16**	6.79*	26.13**	52.95**	44.47**

N = 311; **p < 0.01, *p < 0.05; two-tailed test.

Table 5 shows that knowledge specificity and learning innovation ability in M2 have a significant positive effect on the knowledge sharing of alliance members (β = 0.0, *p* < 0.05; β = 0.0, *p* < 0.01). In both M3 and M5, knowledge specificity and learning innovation ability have a significant positive effect on innovation performance (β = 0.141, *p* < 0.05; β = 0.370, *p* < 0.01). But when the knowledge sharing of alliance members enters the regression model, the influence of knowledge specificity on innovation performance is no longer significant (M4, β = 0.053, n.s.). However, learning innovation ability still has a significant impact on innovation performance, but it has declined (M6, β = 0.289, *p* < 0.01); at the same time, the knowledge sharing of alliance members has a significant impact on innovation performance (β = 0.354, *p* < 0.01; β = 0.283, *p* < 0.01). These findings show that knowledge sharing among alliance members plays a complete mediating role in the influence of knowledge exclusivity on knowledge technological innovation and plays a partial mediating role in the impact of learning innovation ability on innovation performance. Therefore, research Hypotheses H6 and H7 are verified.

**FIGURE 2:**
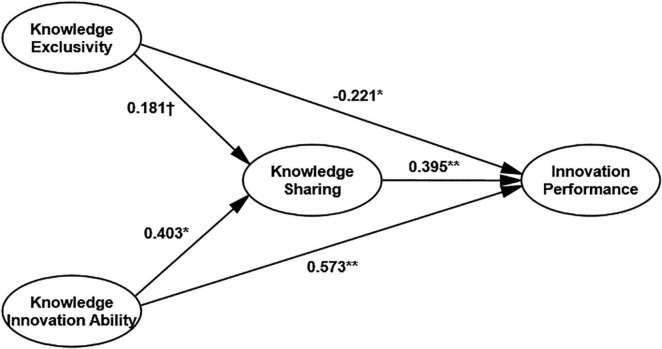
Results of the mediating effect of knowledge sharing on the impact of member ability on innovation performance. **p < 0.01, *p < 0.05, ^†^*p* < 0.10.

The structural equation modeling method is used to test further the mediating role of alliance members’ knowledge sharing in the effect of member ability on alliance performance. Knowledge specificity and learning and innovation ability are independent variables, knowledge sharing among alliance members is an intermediary variable, and innovation performance is an outcome variable, as shown in Figure 2. (*x*^2^/*df* = 1.678; RMSEA = 0.047; IFI = 0.933; CFI = 0.932. *N* = 311; ^**^p < 0.01, *p < 0.05, †*p* < 0.10).

#### Mediating Role of Member Knowledge Sharing in the Effect of Member Relationships on Alliance Innovation Performance

This section uses a linear regression method to test the mediating role of alliance member knowledge sharing in the impact of alliance membership on innovation performance. Table 6 presents the test results of linear regression.

**TABLE 6:** The mediating effect of member knowledge sharing on the impact of member relationship on innovation performance.

Independent variable	Dependent variable: knowledge sharing		Dependent variable: innovation performance			
	M1	M2	M3	M4	M5	M6
Gender	–0.033	–0.022	–0.068	–0.062	–0.079	–0.070
Age	–0.190**	–0.100	–0.183**	–0.149**	–0.205**	–0.160**
Working time	0.047	0.063	–0.110*	–0.123*	–0.081	–0.103*
Cooperative spirit		0.257**	0.338**	0.248**		
Trust level		0.172**			0.308**	0.230**
Knowledge sharing				0.292**		0.310**
R^2^	0.036	0.153	0.201	0.276	0.183	0.270
ΔR^2^	0.036	0.116	0.108	0.183	0.090	0.177
ΔF	3.87*	20.97**	41.46**	38.44**	33.89**	37.09**

N = 311; **p < 0.01, *p < 0.05; two-tailed test.

Table 6 shows that the cooperative spirit and trust level in M2 has a significantly positive effect on the knowledge sharing of alliance members (β = 0.0, *p* < 0.05; β = 0.0, *p* < 0.01). Both the spirit of cooperation and the level of trust in M3 and M5 significantly affect innovation performance (β = 0.338, *p* < 0.01; β = 0.308, *p* < 0.01). When the knowledge sharing of alliance members enter the regression model, the influence of cooperation spirit and trust level on innovation performance is still significant but has declined (M4, β = 0.248, *p* < 0.01; M6, β = 0.230, *p* < 0.01). At the same time, knowledge sharing among alliance members has a significant impact on innovation performance (β = 0.292, *p* < 0.01; β = 0.310, *p* < 0.01). This finding shows that knowledge sharing among alliance members partially mediates cooperation spirit and trust level in innovation performance. Therefore, research Hypotheses H8 and H9 are verified.

**FIGURE 3:**
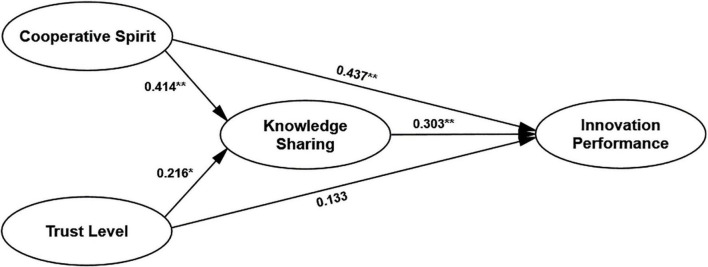
The mediating role of alliance member knowledge sharing in the impact of membership on innovation performance. **p < 0.01, *p < 0.05.

The structural equation modeling method is used to test further the mediating role of alliance member knowledge sharing in the effect of membership on alliance performance. Cooperative spirit and trust level are independent variables, knowledge sharing among alliance members is an intermediary variable, and innovation performance is an outcome variable, as shown in Figure 3. *x*^2^/*df* = 2.166; RMSEA = 0.061; IFI = 0.913; CFI = 0.911. *N* = 311; ^**^p < 0.01, *p < 0.05).

## Conclusion and Discussion

### Conclusion

eSports industry knowledge alliances have become the main structural form and source of innovation for eSports enterprises. However, both in practice and theory, these alliances are still a relatively new concept. Based on previous scholars’ research on innovation performance, the present research explores the influence of alliance member ability and membership relationship on alliance innovation performance utilizing a questionnaire survey and analyses the mediating role of knowledge sharing. The reliability and validity tests of the measurement scale and the whole model were tested using SPSS 24.0 software and AMOS 24.0 software. Through theoretical analysis and empirical research, the following conclusions are obtained. (1) Member ability, member relationships, and member knowledge sharing significantly impact the innovation performance of eSports industry knowledge alliances. (2) Member knowledge sharing plays a mediating role in the effect of member ability and membership relationship on the innovation performance of eSports industry knowledge alliances.

### Theoretical Contributions

Member ability, member relationships, and member knowledge sharing have a significantly positive impact on the innovation performance of eSports industry knowledge alliances. Membership ability is reflected mainly in knowledge specificity and learning ability. When selecting members of the eSports industry knowledge alliance, priority should be given to choosing enterprises or organizations with robust learning and innovation capabilities and existing knowledge that complements other alliance members (Camisón and Villar-López, 2014; Najafi-Tavani et al., 2018). Membership is reflected mainly in the cooperative spirit and trust level of members, which are the basis for cooperation among alliance members (Jones et al., 2018). The theoretical assumptions that motivation induces behavior and that willingness guides action are also applicable from studying individual behavior to organizational behavior. Knowledge sharing is the foundation of promoting eSports knowledge development (Singh et al., 2021). eSports industry knowledge alliances are not enterprise organizations established based on equity but rather loose alliances based on the development of eSports knowledge.

Knowledge sharing among alliance members plays a complete mediating role in the influence of knowledge exclusivity on technological innovation. It also plays a partial mediating role in the impact of learning innovation ability on innovation performance. Knowledge sharing among alliance members plays a partial mediating role in the influence of cooperation spirit and trust level on innovation performance. Member ability and membership relationships further affect alliance performance by affecting the knowledge sharing of eSports industry knowledge alliances. Knowledge sharing transmits information to the other party and digests and absorbs the shared knowledge, integrates it into its knowledge structure, and develops new knowledge capabilities (Ganguly et al., 2019). eSports industry knowledge alliances are common for eSports companies and organizations to expand eSports knowledge and innovate eSports products and formats.

### Practical Contributions

#### Carefully Select eSports Industry Knowledge Alliance Members

This research has confirmed the importance of the selection of eSports industry knowledge alliance members. How to identify and selecting potential partners in practice is the prerequisite for the sustainable development of eSports industry knowledge alliances. Therefore, the selection of eSports industry knowledge alliance members should fully consider the following aspects.

The selection of alliance members needs to consider the complementarity of knowledge. The investigation of the knowledge characteristics of the eSports industry in this paper proves that the knowledge exclusivity among eSports industry knowledge alliance members has a positive impact on alliance performance. Therefore, eSports industry knowledge alliances should select members with different knowledge categories from the existing members and consider the mutual matching of knowledge systems among members when establishing cooperation. Through the differentiated resources invested in by all parties, various members provide alliances with entirely different types of resources or skills.

Organizations with solid learning abilities should be selected as alliance members. The learning ability of eSports industry knowledge alliances determines the effectiveness of eSports knowledge innovation. Therefore, members should be chosen from those organizations that pursue technological progress and maintain a certain speed of knowledge updating. The partners of eSports industry knowledge alliances are not necessarily limited to those inside the eSports industry. They can exist outside the eSports industry, those with strong technical capabilities, or cross-industry partners who occupy a substantial market according to the alliance’s knowledge innovation direction.

#### Build Harmonious Relationships Among Alliance Members

This study proves that alliance membership positively impacts the performance of knowledge alliances in the eSports industry. The influence of member relationships on performance is much stronger than member ability. Based on this, this paper proposes the following recommendations.

During the member selection stage, the similarity of organizational culture among members should be fully considered. Therefore, in selecting members, especially core members, it is necessary to fully consider relevant enterprises or organizations with an innovative spirit in the organizational culture. Members must recognize the league’s eSports knowledge innovation activities rather than profit from joining the company (Prajogo and Ahmed, 2006).

Mutual trust and teamwork within the alliance and among its members should be encouraged. It is necessary to negotiate to determine the ownership of intellectual property rights within the alliance and the sharing ratio of eSports knowledge innovation achievements to reduce the risk of learning among members.

#### Facilitate Broad Knowledge Sharing Among Consortium Members

For the eSports industry knowledge alliance, attention should be paid to the simultaneous advancement of knowledge sharing in various aspects. As an emerging industry, the eSports industry is more likely to be deeply integrated with other sectors in the future. eSports industry knowledge alliances should promote the deep integration of eSports knowledge and traditional business formats to expand the profit space of the eSports industry. However, the eSports intellectual property is its core resource for any member, and the company hopes to monopolize the cooperation results. Therefore, how to balance the relationship between knowledge possession and knowledge flow direction in this contradictory state and promote cooperation among members, especially knowledge cooperation, has become one of the most critical tasks.

### Limitations and Directions for Future Research

There are significant differences between Chinese and Western cultures, and the connotations of variables may be inconsistent against different cultural backgrounds. This study follows the concepts and measurements of Western scholars on member ability, member relationships, and knowledge sharing. Still, it lacks a comparison of the connotations for these variables against the backgrounds of Chinese and Western cultures. Therefore, the comparative analysis of the above variables in different cultural contexts and measurement scales of member ability, member relationships, and knowledge sharing in local Chinese culture needs to be developed.

In the future development of research, the following issues should be considered and explored:

(1) Empirical research on different types of eSports companies needs to be conducted in-depth. eSports industry knowledge alliances cover almost all eSports companies, including eSports technology developers, eSports game developers, eSports game operators, eSports competition organizers, operators, etc. These eSports companies or organizations that join eSports industry knowledge alliances have different motivations and play different roles. As part of the leading eSports industry knowledge alliance, which eSports enterprise is the most efficient? Which eSports company is the most critical player in the league and critically impacts performance? These issues require in-depth research.

(2) The impact of alliance members’ different attitudes toward the effects of eSports innovation on alliance performance needs further research. eSports companies or organizations have different attitudes toward eSports innovation. According to the perspectives of eSports enterprises toward innovation, it is necessary to study their impact on the performance of knowledge alliances in the eSports industry.

(3) The impact of the eSports industry knowledge alliance structure on performance must be further verified. Due to the rapid development of the eSports industry, eSports industry knowledge alliances cover an increasing number of types of organizations, and they become alliance members through different channels. How to describe these structural forms, quantify them and incorporate them into the performance analysis model to find the most effective form of knowledge alliance in the eSports industry requires further in-depth research.

## Data Availability Statement

The original contributions presented in the study are included in the article/supplementary material, further inquiries can be directed to the corresponding author/s.

## Author Contributions

LY designed the study and analyzed the data. YZ discussed the results. MY drafted the manuscript. All authors have agreed to be accountable for all aspects of the manuscript in ensuring that questions related to the accuracy or integrity of any part of it are appropriately investigated and resolved.

## Conflict of Interest

The authors declare that the research was conducted in the absence of any commercial or financial relationships that could be construed as a potential conflict of interest.

## Publisher’s Note

All claims expressed in this article are solely those of the authors and do not necessarily represent those of their affiliated organizations, or those of the publisher, the editors and the reviewers. Any product that may be evaluated in this article, or claim that may be made by its manufacturer, is not guaranteed or endorsed by the publisher.
